# Intensity-modulated stereotactic radiosurgery for arteriovenous malformations: guidance for treatment planning

**DOI:** 10.1186/1748-717X-9-73

**Published:** 2014-03-10

**Authors:** Marcus Sonier, Ermias Gete, Chris Herbert, Michael McKenzie, James Murphy, Vitali Moiseenko

**Affiliations:** 1 British Columbia Cancer Agency, Vancouver Centre, 600 West 10th Avenue, Vancouver, BC V5Z 4E6, Canada; 2 University of California San Diego, Rebecca and John Moores Comprehensive Cancer Center, 3855 Health Sciences Drive, La Jolla, CA 92093-0843, USA; 3 Odette Cancer Centre, Sunnybrook Health Sciences Centre, 2075 Bayview Avenue, T-wing TG217, Toronto, ON M4N 3M4, Canada

**Keywords:** Stereotactic radiosurgery, Intensity modulated radiotherapy (IMRT), Arteriovenous malformation (AVM), Treatment planning, Patient stratification

## Abstract

**Background:**

Stereotactic Radiosurgery (SRS) is a common tool used to treat Arteriovenous Malformations (AVMs) in anatomical locations associated with a risk of surgical complications. Despite high rates of clinical effectiveness, SRS carries a risk of toxicity as a result of radiation injury to brain tissue. The use of intensity-modulated radiotherapy (IMRT) has increased because it may lead to improved PTV conformity and better Normal Tissue (NT) sparing compared to 3D Conformal Radiotherapy (3DCRT). The aim of this study was twofold: 1) to develop simple patient stratification rules for the recommendation of IMRT planning strategies over 3DCRT in the treatment of AVMs with SRS; and 2) to estimate the impact of IMRT in terms of toxicity reduction using retrospectively reported data for symptomatic radiation injury following SRS.

**Methods:**

Thirty-one AVM patients previously treated with 3DCRT were replanned in a commercial treatment planning system using 3DCRT and static gantry IMRT with identical beam arrangements. The radiotherapy planning metrics analyzed included AVM volume, diameter, and volume to surface area ratio. The dosimetric endpoints analyzed included conformity index improvements and NT sparing measured by the maximum NT dose, and the volume of surrounding tissue that received 7Gy and 12Gy.

**Results:**

Our analysis revealed stratified subsets of patients for IMRT that were associated with improved conformity, and those that were associated with decreased doses to normal tissue. The stratified patients experienced an improvement in conformity index by −6-68%, a reduction in the maximum NT dose by −0.5-12.3%, a reduction in the volume of NT receiving 7Gy by 1-8 cc, and a reduction in the volume of NT receiving 12Gy by 0–3.7 cc. The reduction in NT receiving 12Gy translated to a theoretical decrease in the probability of symptomatic injury by 0–9.3%.

**Conclusions:**

This work indicates the potential for significant patient improvements when treating AVMs and provides rules to predict which patients are likely to benefit from IMRT.

## Background

Arteriovenous Malformations (AVMs) can be treated using a variety of techniques including surgery, embolization, or Stereotactic Radiosurgery (SRS), any of which can obtain optimal results. Among these modalities, SRS is often the treatment of choice for AVMs in anatomical locations associated with a risk of surgical complications [1]. Through the application of SRS, complete nidal obliteration is the desired curative outcome, and typically takes up to 2–3 years to manifest with a success rate of 60-90% [1-8]. Additionally, Normal Tissue (NT) toxicity may arise from SRS with complications reported to occur in 3-7% of patients [1,3,6,9-12].

AVM obliteration depends principally on the minimum dose to the Planning Target Volume (PTV), typically greater than 18Gy [8,13-15]. Two distinct dosimetric factors have consistently shown to be strong predictors of symptomatic injury: V_x_, volume receiving × Gy or more, and conformity of the dose distribution encompassing the PTV [4,6,10,12,16-18]. A recent Quantitative Analysis of Normal Tissue Effects in the Clinic (QUANTEC) report summarized toxicity data from a selection of studies and demonstrated that V_10_ and V_12_ are particularly strong and consistently observed predictors of toxicity, with V_12_ serving as a predictor of choice [18]. Similarly, a strong correlation between various volumetric endpoints and subsequent brain injury has also been reported at lower doses such as V_7_[17]. Radiation doses with SRS plans balance the risk of symptomatic brain injury with the likelihood of nidus obliteration.

Optimizing radiation plans to minimize the risk of toxicity is particularly difficult to achieve for complex-shaped AVMs. Intensity Modulated Radiotherapy (IMRT) has been proposed as a means to improve plan conformity and thereby reduce the risk of complications [13]. Despite the potential benefit of IMRT, its implementation is costly and resource-intensive. In addition to planning, patient-specific Quality Assurance (QA) is required, usually involving ion chamber measurements at the isocenter and a comparison of planned and delivered fluence maps. Furthermore, with the introduction of image guided frameless SRS, it has become common practice to treat stereotactic patients with IMRT.

Given the resources involved in using IMRT, we sought to develop a decision tool that identified the subset of AVM patients who would benefit the most from IMRT. The specific aims of this study are twofold: 1) to develop simple patient stratification rules for the recommendation of IMRT planning strategies over 3DCRT in the treatment of AVMs with SRS, and 2) to estimate the impact of IMRT in terms of toxicity reduction using retrospectively reported data for symptomatic radiation injury following SRS.

## Methods

CT, MR, and Angiogram image sets for thirty-one AVM patients previously treated at the BC Cancer Agency were used in this study. These image sets were transferred to an up-to-date version of the iPlan (BrainLAB AG, Heimstetten, Germany) treatment planning system software for use with the BrainLab microMLC. The CT and Angiogram images were localized and fused to the MR images, followed by manual contouring of each patient’s AVM by an oncologist specializing in SRS. Next, a PTV was constructed by expanding the AVM contour by a 1 mm margin in 3-dimensions, and treatment plans utilizing 3DCRT and static gantry IMRT were produced as described below. The study was approved by the institutional ethics committee.

### A. 3D Conformal radiotherapy

In 3DCRT, plans used for patient treatment were consulted as a guideline for the positioning and number of beams. With these guidelines, single isocenter treatment plans were produced with PTV coverage as the primary objective while respecting dose to Organs At Risk (OARs). Dose was prescribed to the isocenter of the PTV. Multi-Leaf Collimator (MLC) shapes were manually adjusted to achieve PTV coverage so that the 80% isodose line encompassed the PTV, minimizing spillage of the 80% isodose to surrounding NT. Multiple fields (typically at least 10) were used to minimize the beam overlap effect and MLC shapes were manipulated manually to achieve the planning objectives. PTV coverage was optimized and verified in the axial, coronal, and sagittal slice views on both CT and MR image sets and in the Dose-Volume Histogram (DVH) view.

### B. Intensity modulated radiotherapy

**Figure 1:**
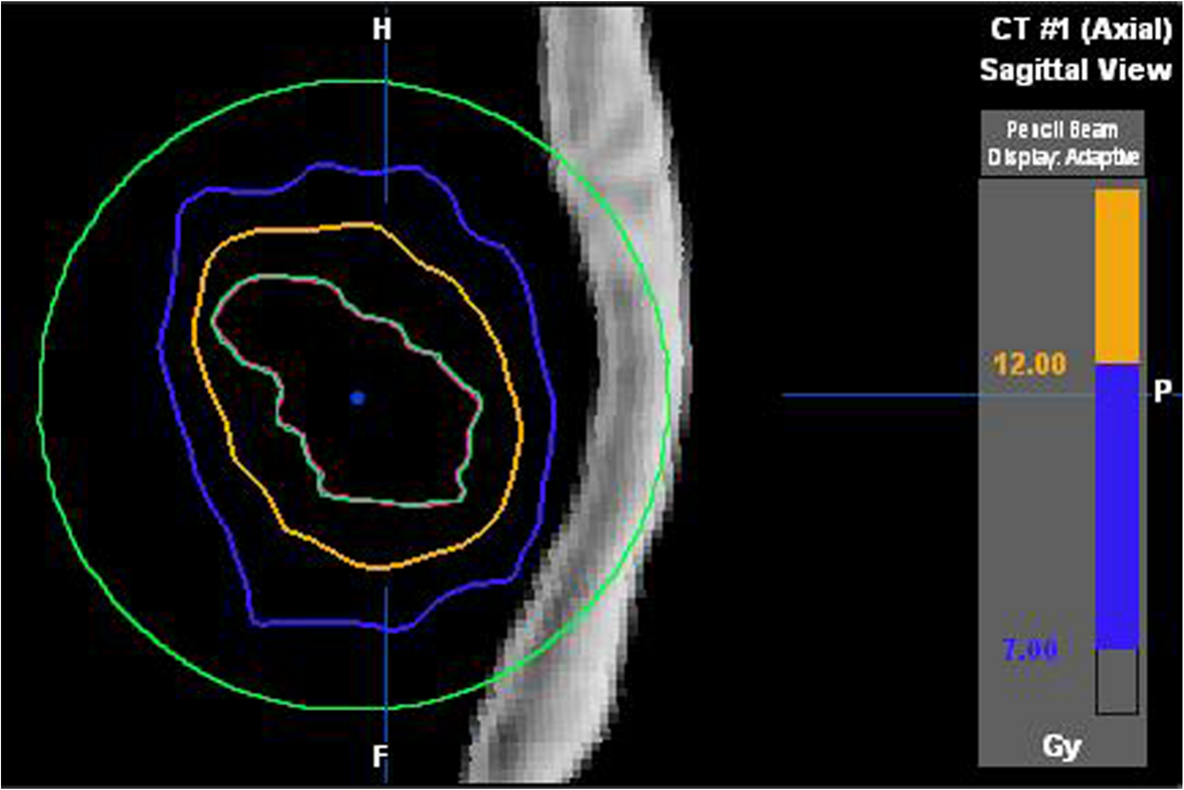
**OAR sphere.** A representative patient’s PTV encompassed by an OAR sphere (bright green). The sphere fully contains the 7Gy isodose (blue) in order to allow IMRT constraints to adequately restrict dose to NT.

The beam arrangement with IMRT was identical to those used with 3DCRT. The dose to the PTV margin, corresponding to the 80% isodose in the 3DCRT plans, was specified as a hard constraint. The dose optimization settings were selected as follows: PTV dose calculation grid size = 1 mm, OAR dose calculation grid size = 2 mm, Beamlet size max = 1 mm, Step-and-Shoot leaf sequencing, and Tongue-and-Groove Optimization for MLC leaf positioning. Initially, a sample plan was generated to determine the volume of NT enclosed by the 7Gy isodose surface. This 7Gy isodose surface was used to construct a normal tissue OAR sphere that fully enclosed the 7 and 12Gy isodose surfaces, with the PTV removed. Figure 1 illustrates the OAR sphere encompassing the target volume. This OAR sphere was used to apply NT constraints at the 7 and 12Gy isodose volumes during IMRT optimization. For each patient, four IMRT plans were generated using utilities available in the iPlan treatment planning system: PTV Only, OAR Low, OAR Medium, and OAR High. PTV Only represented an IMRT plan that optimized solely with the PTV, ignoring the surrounding OAR. OAR Low, Medium, and High represented plans that attempted to limit dose to the OAR sphere with increasing weighting (from low to high). With the OAR Low, Medium, and High plans, the NT constraints were applied at the dose-volumes of interest (7 and 12Gy) and were patient specific, depending on the initial dose the NT received as a result of the PTV's size and shape. Therefore, the NT constraints were set through an iterative process such that, through the IMRT optimization algorithm, maximum NT sparing was achieved without sacrificing PTV coverage. With each IMRT plan, greater than 99.9% of the PTV volume received the prescribed dose.

### C. Plan comparisons

The AVM metrics obtained from each patient included the volume of the PTV, the maximum PTV diameter, and the ratio of the volume of the PTV to the surface area of the PTV (Vol./SA). Treatment planning systems typically do not provide tools to calculate the surface area. Our goal was also to provide a solution which can be implemented using tools routinely available in planning systems, such as expanding volumes by a margin. The surface area was therefore estimated from the change in volume from the 1 mm expansion of the AVM to the PTV divided by 1 mm. The accuracy of this approximation improves as the expansion margin decreases. However, by trial-and-error method we established that that margins smaller than the slice thickness lead to irregularities on the expanded contour. Conversely, a larger margin makes this method inaccurate for complex-shaped lesions. The 1 mm margin has been selected as an acceptable compromise. In the narrative below SA is used in the context of the used approximation. The Vol./SA measurement attempted to characterize the complexity of the shape of the PTV. Shapes well approximated by a sphere have Vol./SA measurements comparable to expected for a sphere with the same volume, while complex shapes have low Vol./SA measurements compared to a sphere with the same volume.

The dosimetric endpoints analyzed included the conformity index which was defined as the volume enclosed by the prescription dose divided by the PTV volume. Additional endpoints included the maximum NT dose, and the volume of NT receiving 7 and 12Gy. Plots were constructed to show the benefits of each of the four IMRT plans compared to 3DCRT plans as measured by each dosimetric endpoint defined above. The individual plots were assessed to find any visual pattern of benefits that clearly indicated potential stratification rules that could be used to isolate patients who would benefit from IMRT. Statistical analysis was then performed via the one-tailed t-test to determine the significance of two aspects of the results: the benefit of PTV Only IMRT vs. OAR Low IMRT for all patients and the patient improvements in the stratified subset vs. the unstratified subset for each dosimetric endpoint. When comparing PTV Only IMRT vs. OAR Low IMRT, all 31 patients were considered whereas when analysing the stratification results, for conformity index, NT max dose, V_7_, and V_12_, the 31 patients were split into two groups (no patients were omitted) following the established stratification rules.

## Results

Patient treatment characteristics are shown in Table 1. The prescribed dose was delivered in a single fraction and varied by the size, shape, and location of the PTV. Thus, larger PTVs received lower dose prescriptions, with a reduced probability of AVM obliteration, as a compromise for avoiding NT complications from irradiating larger volumes of normal brain tissue. Anatomic locations of the AVMs varied widely throughout the brain, occurring next to relatively deep and important structures such as the brainstem, optic nerves, and thalamus as well as out towards the brain’s periphery: in the frontal, temporal, parietal, and occipital regions.

**Table 1:** Patient summary

**Patient summary**	
Number of Patients	31
PTV Diameter (cm)	Range: 0.99-5.38
	Median: 2.71
PTV Volume (cc)	Range: 0.334-26.720
	Median: 2.666
Prescribed Dose (Gy)	Range: 12–25
	Median: 20

Summary of patient data concerning lesion characteristics and prescribed treatment dose.

**Figure 2:**
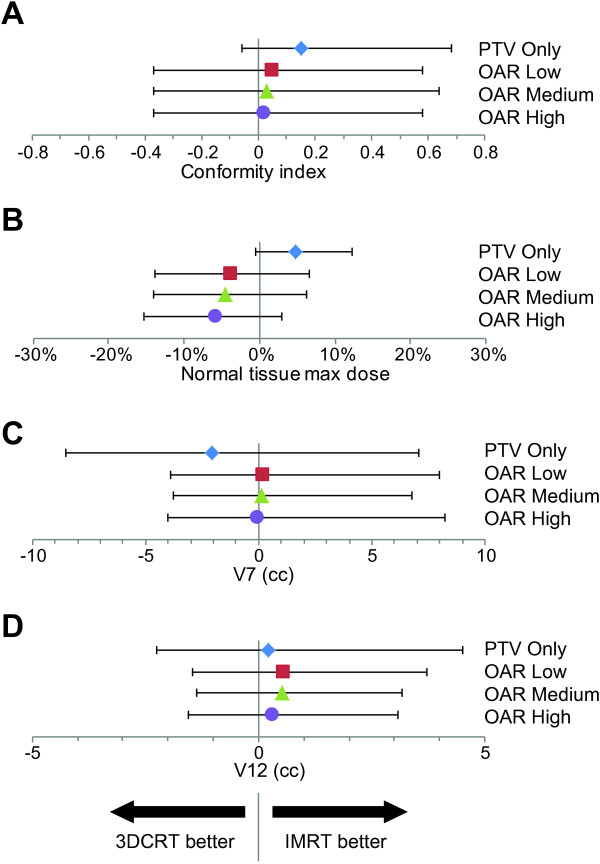
**Endpoint Comparisons for 3DCRT vs. IMRT.** Points represent the median difference between IMRT and 3DCRT plans for conformity index **(A)**, normal tissue maximum dose **(B)**, V_7_**(C)**, and V_12_**(D)**. For each patient, four different IMRT plans were generated including PTV Only (blue diamond), OAR Low (red square), OAR Medium (green triangle), and OAR High (purple circle). Positive values represent circumstances where IMRT outperforms 3DCRT, and negative values represent circumstances where 3DCRT outperforms IMRT. Error bars represent the range of values.

First, we compared the difference between 3DCRT and each of the four IMRT plans for all patients in this study. These results are shown in Figure 2 with the dosimetric endpoints of conformity index, NT max dose, V_7_, and V_12_ depicted in A-D, respectively. With conformity index, each of the IMRT plans on average produced more conformal plans than 3DCRT, and of the IMRT techniques, PTV Only had the largest improvement over 3DCRT. With NT max dose, only the PTV Only IMRT plan had lower maximum dose than 3DCRT. OAR Low, Medium, and High IMRT plans more often had higher maximum NT doses. With the V_7_ dosimetric endpoint, the PTV Only and OAR High IMRT plans increased the NT volume receiving 7Gy, whereas the OAR Low and OAR Medium plans decreased the NT volume receiving 7Gy. With the V_12_ dosimetric endpoint, each of the IMRT plan types on average reduced the NT volume receiving 12Gy, compared to 3DCRT, with the OAR Low and OAR Medium plans providing the greatest overall benefit.

**Figure 3:**
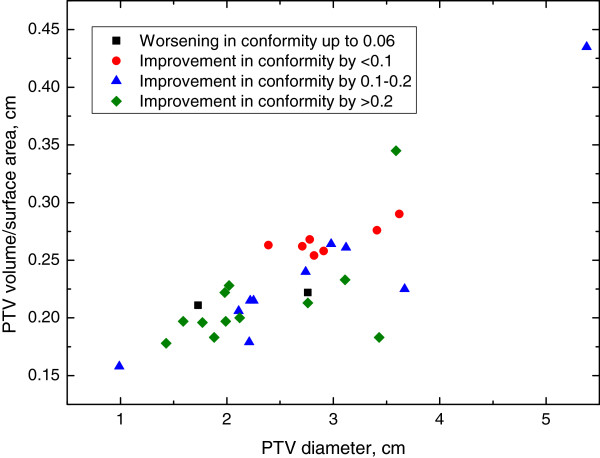
**Conformity Index (PTV Only IMRT).** Distribution of patient improvements regarding PTV conformity when comparing PTV Only IMRT to 3DCRT. Patient stratification into groups, depending on the benefit received from IMRT, is readily observed when plotting Vol./SA vs. PTV Diameter while alternate plots do not clearly indicate a stratified subset of patients with improved treatment parameters.

**Figure 4:**
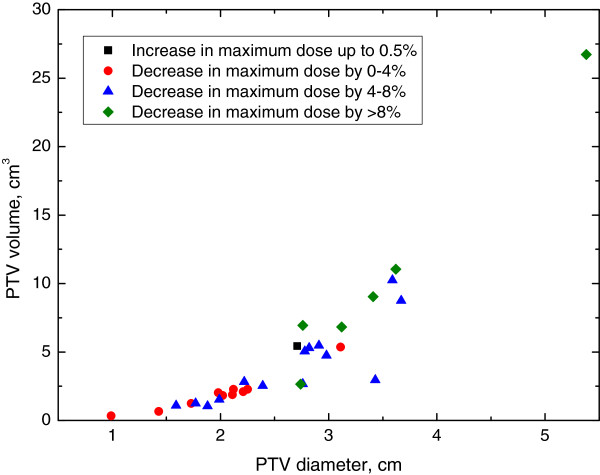
**Normal Tissue Maximum Dose (PTV Only IMRT).** Distribution of patient improvements regarding the max dose to NT when comparing PTV Only IMRT to 3DCRT. Patient stratification into groups, depending on the benefit received from IMRT, is readily observed when plotting PTV Volume vs. PTV Diameter while alternate plots do not clearly indicate a stratified subset of patients with improved treatment parameters.

**Figure 5:**
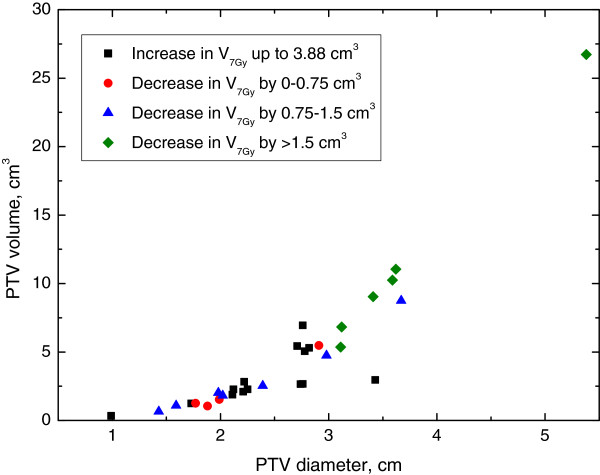
**Normal Tissue 7Gy Isodose Volume (OAR Low IMRT).** Distribution of patient improvements regarding the volume of NT receiving 7Gy when comparing OAR Low IMRT to 3DCRT. Patient stratification into groups, depending on the benefit received from IMRT, is readily observed when plotting PTV Volume vs. PTV Diameter while alternate plots do not clearly indicate a stratified subset of patients with improved treatment parameters.

**Figure 6:**
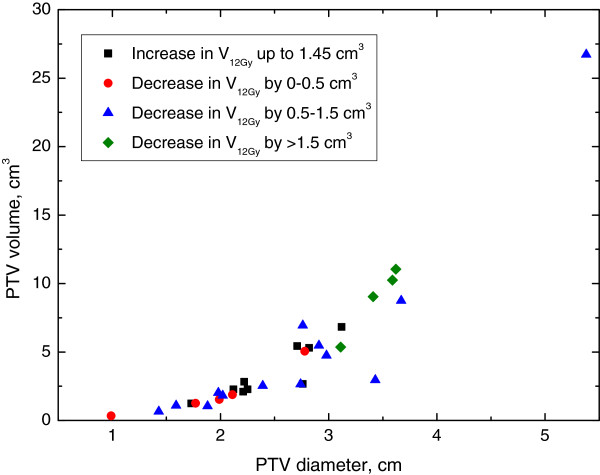
**Normal Tissue 12Gy Isodose Volume (OAR Low IMRT).** Distribution of patient improvements regarding the volume of NT receiving 12Gy when comparing OAR Low IMRT to 3DCRT. Patient stratification into groups, depending on the benefit received from IMRT, is readily observed when plotting PTV Volume vs. PTV Diameter while alternate plots do not clearly indicate a stratified subset of patients with improved treatment parameters.

Next, we explored whether characteristics of the AVM were associated with circumstances where IMRT was consistently superior to 3DCRT. Figure 2A-D demonstrates select 2×2 plots that show conditions where IMRT outperforms 3DCRT for each of the dosimetric endpoints analyzed. Figure 3 shows the distribution of improvements in conformity index for IMRT compared to 3DCRT with the PTV Only IMRT plan. IMRT was consistently associated with an improved conformity index when Vol./SA <0.25 cm and PTV diameter <2.25 cm. Figure 4 shows the distribution of reduction in maximum NT dose for IMRT compared to 3DCRT with the PTV Only IMRT plan. In this case, patients with PTV diameters greater than 2.5 cm appear to benefit the most from IMRT. Figures 5 and 6 show the distribution of improvements in V_7_ and V_12_, respectively, with OAR Low IMRT plans. With V_7_ (Figure 5), patients whose PTV volumes are >5 cc and PTV diameters >3 cm had the largest degree of NT sparing with IMRT. With V_12_ (Figure 6), patients with PTV diameters >3 cm received the largest NT sparing with IMRT. Finally, Table 2 summarizes the information contained in Figures 3, 4, 5, and 6 by presenting simple stratification rules that demonstrate the AVM characteristics where IMRT outperforms 3DCRT.

**Table 2:** PTV stratification rules

		**Characteristics of potential benefit with IMRT**			
		**PTV conformity**	**NT max dose**	**7Gy isodose vol.**	**12Gy isodose vol.**
PTV stratification rules	Diameter	<2.25 cm	>2.5 cm	>3.0 cm	>2.9 cm
	Volume	N/A	N/A	>5.0 cc	N/A
	Vol./SA	<0.25 cm	N/A	N/A	N/A
Predicted improvement		−0.06-0.68	−0.5-12.3 %	1.0-7.952 cc	−0.024-3.704 cc

PTV stratification rules and predicted improvements for patient benefit with the use of IMRT for the treatment of AVMs. The predicted improvements are the ranges obtained within this study for patients whose lesion characteristics satisfy the rules concerning each dosimetric parameter stated above. Within these rules, the boxes labeled “N/A” for the corresponding PTV characteristic depict no clear boundary defining patient benefit resulting in the inability for patient stratification to be achieved.

Finally, statistical analysis revealed p-values of <0.001 for conformity index, NT max dose, and V_7_ while V_12_ had a p-value of 0.024 when comparing PTV Only IMRT benefits against OAR Low IMRT benefits. Comparing stratified patients vs. unstratified patients for the plan type of choice for each dosimetric endpoint revealed p-values of 0.023, 0.400, 0.001, and 0.002 for conformity index (PTV Only IMRT), NT max dose (PTV Only IMRT), V_7_ (OAR Low IMRT), and V_12_ (OAR Low IMRT), respectively.

## Discussion

The first intent of this study was to identify characteristics of an AVM that predicted improvement with IMRT compared to 3DCRT. This allows a treatment team to identify patients who are most likely to benefit from IMRT. This study also demonstrates the tradeoffs of IMRT and 3DCRT. Stratification criteria such as these allow a physician to choose which dosimetric endpoint carries the most importance for a given patient, and then use the characteristics of the individual AVM to determine whether IMRT is likely to generate a superior plan compared to 3DCRT. This could potentially reduce treatment time, reduce resource use, and improve plan quality which impacts patient care.

The safe and effective treatment of AVMs with SRS balances dose to the PTV with dose to surrounding normal tissues. The importance of dose to the periphery of the AVM was demonstrated by Herbert *et al.* who found that dose to the PTV margin >20Gy was strongly associated with nidus obliteration [14]. Because the incidence of symptomatic brain injury correlates with dose to surrounding NT, plan conformity is of the utmost importance. This result has also been validated in a study by Friedman *et al.* who found that improved PTV conformity correlates with a reduced incidence of complications [10]. Thus, the choice of the optimal radiation planning modality to improve the conformity index can significantly reduce the probability of SRS induced complications. In our study, this level of improvement in treatment plans was found to occur more so in patients with a large PTV surface area compared to their volume (small Vol./SA ratio, <0.25 cm), and small PTV diameters (<2.25 cm).

In addition to the importance of conformity index, maximum dose to NT should not be overlooked. Unlike other cancerous stereotactic targets, the target volume in an AVM may contain regions of normal brain tissue intermittently spaced between the blood vessels. The normal tissue surrounding and within the PTV is at risk of toxicity with SRS. Therefore, it is desirable to minimize hotspots in these areas by maintaining the dose to the PTV as close to the prescribed dose as possible. The IMRT plans in this study accomplished this by reducing the max dose to NT and the PTV, increasing the steepness of the dose fall off gradient at the edges of the PTV, and reducing and distributing any hot spots diffusely throughout the PTV compared to 3DCRT. This reduction in dose to volumes potentially containing NT could also reduce the risk of symptomatic brain injury. Along the lines of maximum dose, the 12Gy isodose volume has been investigated by numerous groups and consistently shown to strongly correlate with symptomatic brain injury. This 12Gy cut-off may not be the only predictor as volumes receiving doses of at least 7Gy among other radiation doses also correlate with a risk of brain injury [17,19]. We found similar stratification rules associated with larger PTV diameters that predict superior plans with IMRT with respect to NT max dose, V_7_, and V_12_. This suggests that reduction in one normal tissue constraint does not come at a cost of increase in another with IMRT planning. To determine the significance of our rules, we performed statistical analysis on the two patient groups separated via stratification for each endpoint. The resultant p-values obtained when considering conformity index, NT max dose, V_7_, and V_12_, for the suggested IMRT plan of PTV Only or OAR Low, amounted to 0.023, 0.400, 0.001, and 0.002, respectively. Thus, the stratification rules given in Table 2 for conformity, V_7_, and V_12_ are statistically significant (p < 0.05) while those for the NT max dose are not; nevertheless, the use of IMRT produced some improvement for all but one patient with this endpoint. This was likely a direct result of the nature of IMRT treatments suggesting the NT max dose would still benefit from IMRT, decreasing patient complication rates.

In a report by QUANTEC, radiation dose-volume effects in the brain were reviewed and complication rates based on the 12Gy isodose volume were analyzed [18]. The investigators of this study concluded that brain toxicity increases rapidly when more than 5-10 cc of NT is irradiated with >12Gy. IMRT achieves a decrease in V_12_ with the stratified patients in Table 2 showing significant NT sparing effects. In fact, the predicted benefits for the 7 and 12Gy isodose volumes of 1.0 - 7.952 cc and −0.024 - 3.704 cc, respectively, translate to considerable decreases in the probability of radiation-induced brain injury post-SRS when interpreting the results from the QUANTEC study [18]. In this publication, probabilities for radiation necrosis as a function of volume irradiated are plotted for a multitude of studies concerning different isodose volumes: 10Gy, 12Gy, and the treatment volume. However, only two data sets relating symptomatic injury to the 12Gy isodose volume are of interest: the Korytko 2006 and the Flickinger 1997 results. Each data set represents substantially different variations in radiation necrosis with the 12Gy isodose volume that, when simplified through averaging the slopes of the linear portions of the plot, amount to a 0.6%/cc and 2.5%/cc increase in incidence of complications for the Flickinger and Korytko studies, respectively. Comparing this with the 12Gy isodose volume decreases attained with IMRT (i.e. multiplying 0.6%/cc and 2.5%/cc with the lower and upper limits on the V_12_ benefits, respectively), the probability of symptomatic radiation injury in the stratified subset of patients may be decreased by 0–9.3%. Although these improvements appear promising, they are simply a projected change in the incidence of brain injury obtained through theoretical calculations and true benefit can only be established with a clinical follow-up study.

Table 3 then translates the stratification rules from Table 2 into simple recommendations for the ideal IMRT planning technique dependent upon the Spetzler-Martin Grade determined at the time of diagnosis [20]. The key aspects of each AVM grade that determine the corresponding IMRT recommendation are size and eloquence. If an AVM is situated in an eloquent part of the brain or is of a small size (diameter < 2.5 cm and small Vol./SA ratio) then PTV Only IMRT is recommended; however, if the AVM is of a large size (diameter >2.5 cm) and not eloquently located then OAR Low IMRT is recommended. While OAR Medium and OAR High plans provided comparable benefits to PTV Only and OAR Low plans in some dosimetric aspects, they routinely contained hotspots in the NT that exceeded realistic restrictions on clinical treatment plans and, as such, are not recommended for patient use. OAR Low plans were found to provide the greatest benefit to patients with large sized AVMs which is due to the larger volume of NT that is irradiated in these plans together with the fact that probability of symptomatic complications following SRS is measured in terms of absolute volumes spared. PTV Only plans, on the other hand, provided improvements in PTV conformity for nearly all patients, regardless of AVM size, which efficiently constrains the prescription dose to the curvature of the PTV and spares adjacent OARs of this high dose. The difference in the mean values for all four dosimetric endpoints between PTV Only and OAR Low IMRT plans were found to be statistically significant (p < 0.05). These benefits provided by the IMRT plans with the lowest weighting on OARs is likely a result of the large of amount of fields that have been utilized (typically >10) for the treatment of small targets. This situation already maximizes the OAR sparing so additional sparing then comes at the cost of clipping the PTV beyond the conformal benefits present in each IMRT plan. Thus, the advantages provided by IMRT in these cases is in improvements in PTV conformity, removing hotspots within NT while improving the dose homogeneity across the PTV, and reducing NT volumes receiving intermediate doses.

**Table 3:** IMRT stratification of Spetzler-Martin grading scheme

**AVM grade**	**Size**	**Eloquence**	**Venous drainage**	**IMRT recommendation**
1	1	0	0	PTV only
2	1	1	0	PTV only
	1	0	1	PTV only
	2	0	0	OAR low
3	1	1	1	PTV only
	2	1	0	PTV only
	2	0	1	OAR low
	3	0	0	OAR low
4	2	1	1	PTV only
	3	1	0	OAR low
	3	0	1	OAR low
5	3	1	1	OAR low

The recommended IMRT plan type per Spetzler-Martin grade combination is shown.

## Conclusions

The work presented in this study indicates the potential for significant conformity improvements with PTV Only IMRT and/or dose reduction to NT at the 7 and 12Gy isodoses with OAR Low IMRT as compared to 3DCRT. Additionally, the likelihood of a patient receiving a particular benefit from IMRT is a function of PTV volume, diameter, and surface area. Using these three characteristics, patients can be stratified into distinct groups contingent upon the extent of benefits received from IMRT. As a result, IMRT improvements over 3DCRT can be readily attained on a case-by-case basis using the associated treatment protocol; thus, reducing the risk of symptomatic injury following radiotherapy.

## Competing interests

The authors declare that they have no competing interests.

## Authors’ contributions

VM conceived the idea. MS generated the patient treatment plans and drafted the manuscript. VM and EG participated in treatment planning procedures and approval. VM, MS, EG, and JM analyzed the data and revised the manuscript. MM provided the clinical goals for treatment planning and participated in IMRT optimization. CH delineated the AVMs and critical structures. All authors read and approved the final manuscript.
